# Cystic Fundic Gland Polyps With High-Grade Dysplasia

**DOI:** 10.14309/crj.0000000000001729

**Published:** 2025-06-16

**Authors:** Aditya Avula, Clive Miranda, Mary Hanson, Aun Shah

**Affiliations:** 1 Department of Internal Medicine, Creighton University, Omaha, NE

## CASE REPORT

Fundic gland polyps (FGPs) are the most common type of gastric polyp and are often found incidentally on endoscopy. While low-grade dysplasia has been reported in sporadic FGPs, with prevalence noted to be around 1%,^1^ we present a case of high-grade dysplasia seen in cystic fundic gland polyps.

**Figure 1.**
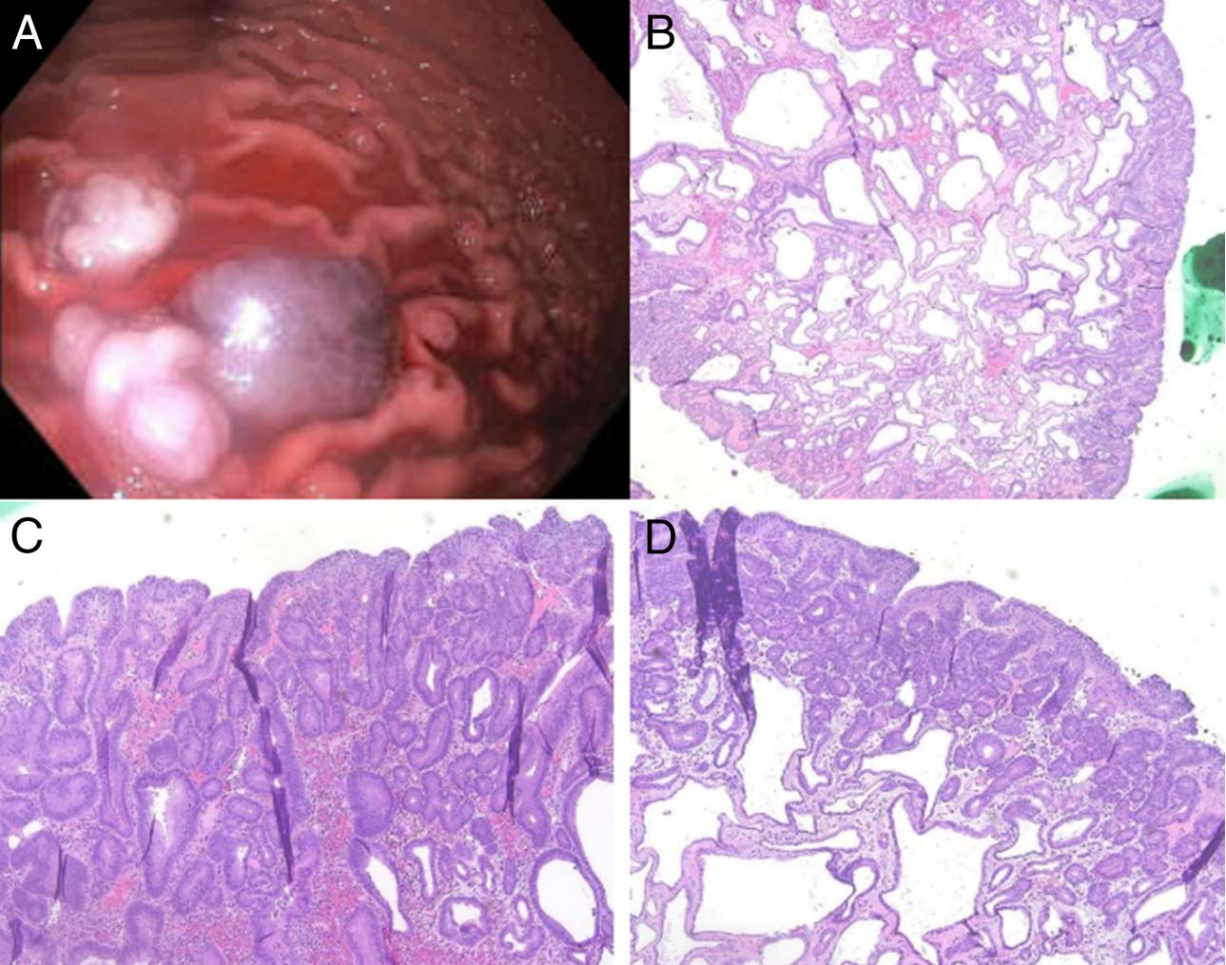
(A) Gastric body with high-grade dysplastic fundic gland polyps. (B) Fundic gland polyp with cystically dilated glands. (C) Surface glands displaying high-grade dysplasia with crowding, frequent mitoses, visible nucleoli, loss of polarity, and complex gland architecture. (D) Dysplastic surface changes with loss of polarity, hyperchromatic nuclei, and crowding.

These images are from a 60-year-old male patient with a significant medical history of Barrett's esophagus on chronic omeprazole therapy who presented for surveillance endoscopy. Three years ago, endoscopic evaluation of the gastric mucosa revealed fundic gland ectasia and overlying mild foveolar hyperplasia consistent with developing fundic gland polyps that was negative for dysplasia. Repeat upper endoscopy showed multiple pedunculated and sessile polyps in the gastric fundus. Three polyps measuring from 10 mm to 20 mm were removed with a hot snare (Figure 1). Resection and retrieval were complete with the placement of 2 hemostatic clips. Biopsies of the gastric fundic polyps revealed cystic fundic gland polyps with high-grade dysplasia (Figure 1). Considering that proton pump inhibitor therapy has been found to be associated with the progression of FGPs, the patient was advised to taper off proton pump inhibitor therapy as tolerated.^2^ While the patient did not have a family history of syndromic polyposis, he was scheduled for further genetic testing, which was not followed through with.

While FGPs are quite common, high-grade dysplasia is rare. This case illustrates the importance of awareness of the malignant potential of FGPs when identified on endoscopy.

## DISCLOSURES

Author contributions: A. Avula and C. Miranda: design of the work; acquisition, analysis, and interpretation of data for the work. A. Avula: drafting and reviewing intellectual content including formations of text and images; submission of final text and imagery. M. Hanson: design of the work; acquisition of pathology images. A. Shah: acquisition of endoscopic images and initial endoscopy, improving text and images; draft revision, and review of final text and imagery. A. Avula is the article guarantor.

Financial disclosure: None to report.

Informed consent was obtained for this case report.
